# Psychoeducation, motivational interviewing, cognitive remediation training, and/or social skills training in combination for psychosocial functioning of patients with schizophrenia spectrum disorders: A systematic review and meta-analysis of randomized controlled trials

**DOI:** 10.3389/fpsyt.2022.899840

**Published:** 2022-09-30

**Authors:** Erin Yiqing Lu, Andy S. K. Cheng, Hector W. H. Tsang, Juan Chen, Samuel Leung, Annie Yip, Jessie Jingxia Lin, Zoe Violet Lam, Wufang Zhang, Miaomiao Zhao, Ning Ma

**Affiliations:** ^1^Department of Rehabilitation Sciences, The Hong Kong Polytechnic University, Kowloon, Hong Kong SAR, China; ^2^Department of Applied Social Sciences, The Hong Kong Polytechnic University, Kowloon, Hong Kong SAR, China; ^3^School of Nursing, The Hong Kong Polytechnic University, Kowloon, Hong Kong SAR, China; ^4^Peking University Sixth Hospital, Peking University Institute of Mental Health, NHC Key Laboratory of Mental Health (Peking University), National Clinical Research Center for Mental Disorders (Peking University Sixth Hospital), Beijing, China

**Keywords:** psychoeducation, motivational interviewing, cognitive remediation training, social skills training, schizophrenia, first-episode psychosis

## Abstract

**Objectives:**

Psychoeducation, motivational interviewing, cognitive remediation training, and social skills training have been found to be effective interventions for patients with schizophrenia spectrum disorders. However, their efficacy on psychosocial functioning when provided in combination remains unclear, compared with all types of control conditions. It would also be meaningful to explore the differences of efficacy in patients with first-episode psychosis (FEP) and those with longer term of illness.

**Methodology:**

The present review followed the guidelines of Preferred Reporting Items for Systematic Reviews and Meta-Analyses (PRISMA). Full-text English journal articles of randomized controlled trials published in the past decade in the databases of PubMed, CINAHL Complete, Embase, and PsycINFO were searched. Included studies were all randomized controlled trials (RCTs) with participants diagnosed with schizophrenia spectrum disorders. The included studies should test combined interventions with at least two components from: psychoeducation, motivational interviewing, cognitive remediation training, and social skills training and incorporate assessment of psychosocial functioning at least at baseline and post-intervention.

**Results:**

Seven studies were included for systematic review, and six of them were eligible for meta-analysis. Five out of the seven studies reported effects on psychosocial functioning that favored combined interventions over any type of control condition. A significant pooled effect was derived from the six studies, SMD = 1.03, 95% CI [0.06, 2.00], *Z* = 2.09, *p* = 0.04, *I*^2^ = 96%. However, the pool effect became insignificant when synthesizing five of the studies with non-FEP patients as participants and four of the studies testing relative effects of combined interventions compared with stand-alone interventions/interventions with one less component. None of the included studies adopted motivational interviewing and only one of the studies worked with FEP patients.

**Conclusion:**

Psychoeducation, cognitive remediation training, and social skills training in combination can effectively enhance psychosocial functioning of patients with schizophrenia spectrum disorders. It is warranted to conduct more RCTs to test the effects of different specific combinations of the above interventions on psychosocial functioning, especially in FEP patients.

## Introduction

Patients with schizophrenia spectrum disorders (SSDs) struggle with not only symptoms of hallucinations, delusions, and disorganized thoughts, but also dysfunctions in their cognition, motivation, and behaviors. The daily living of patients with SSDs is severely disrupted by their deficits in psychosocial functioning. The worldwide estimated population of patients with SSDs in 2019 was approximately 21 million, and the lifetime prevalence of schizophrenia was 0.3–0.7% (1). Treatments for schizophrenia aim not only for eliminating symptoms, but also for enhancing psychosocial functioning. Medication is a primary treatment option that has proven its effectiveness in managing symptoms and improving physical and cognitive functioning (2, 3), but side effects of medication, such as sleepiness, insomnia, and dry mouth, can interfere with daily functioning and reduce quality of life (4). Psychosocial interventions have been recognized as important adjunct treatments for patients with SSDs (2). According to the stress-vulnerability model, patients should be supported and empowered for adherence with treatment and better coping with daily stress, which in turn, can result in better prognosis (5). Hence, a group of psychosocial interventions have been developed and applied to compensate patients’ deficits in motivation, cognition, and behaviors. These interventions aim to facilitate patients’ treatment with antipsychotics and psychosocial rehabilitation, and they mainly include psychoeducation, motivational interviewing, cognitive remediation training, and social skills training.

Psychoeducation is an approach to delivering knowledge about schizophrenia and its treatments to patients and their family members, and it also enables collaborative relationships between health professionals and patients when they work toward recovery (2). Psychoeducation involves a learning process which will bring changes in attitudes, cognition, and behavior (6). Based on a series of meta-analyses, patients who received psychoeducation had a lower relapse and readmission rate than a control group with standard care did, and also that psychoeducation has resulted in higher satisfaction with mental health services and a higher quality of life (6).

Motivational interviewing is used to enhance patients’ willingness to adhere to treatment and make behavioral changes for recovery and relapse prevention (7). Patients’ treatment motivation has been shown to improve after motivational interviewing (8), and compared with a sham control, patients with SSDs who received motivational interviewing have shown higher adherence to intervention (9).

Neurocognitive training is a major type of cognitive remediation training and has the aim of improving cognitive functioning, such as working memory, attention, planning, and executive functions, through learning and practice (2). It is common to develop and adopt computerized programs to train neurocognition, and a meta-analysis has concluded that such computerized training could significantly improve attention and working memory of patients with SSDs (10).

Another major type of cognitive remediation training focuses on social cognition, such as the theory of mind, emotional processing, and empathy (11). Recent systematic reviews have supported the notion that social cognition training is effective in improving the cognitive and affective theory of mind (12), facial emotion identification (13), and social competence (14).

Recent cognitive remediation trainings have been intended to improve metacognition, the cognitive process of “thinking about thinking” (15). Improvement in metacognition was found to co-occur with enhanced neurocognition and social cognition (16), supporting the importance of metacognition as a treatment target. Computerized cognitive remediation programs have been developed and evaluated to enhance several metacognitive sub-functions. For example, a computerized program, “Mybraintraining,” was augmented with modules on metacognition training, and it was found to significantly reduce metacognitive functions of overconfidence and jumping to conclusion (17). Another study reported the effectiveness of a metacognitive-based cognitive remediation program, CIRCuiTS, and it could significantly enhance metacognitive knowledge and metacognitive regulation (18).

Social-skills training is a type of behavior therapy that teaches patients with SSDs the skills they need for communication, relationship building, independent living, and the like (19). A recent Cochrane systematic review has concluded that social skills training was more effective than standard care to improve social functioning, lower relapse rate, and enhance quality of life for patients with SSDs (20).

All the above approaches mainly target different psychological functions. However, it is important to recognize the interconnections among the psychological functions, including motivation, cognition, and behavioral skills. Firstly, the role of motivation in the process of cognitive remediation and learning of social skills should be recognized. According to self-determination theory, people are driven to behavioral changes when such actions are intrinsically rewarding or linked to external goals (21). Choi and Medalia (22) have found that patients with high intrinsic motivation at baseline could largely and significantly benefit from a vocational training. A meta-analytic review also found that the association between motivation and functionality in patients with SSDs was significantly stronger than the association between neurocognition and functionality (23). Hence, it is necessary to enhance patients’ motivation for engagement and adherence through psychoeducation or motivational interviewing before they can be significantly benefited from cognitive remediation training or social skill training.

Secondly, the enhancement in cognition function and social skills appeared to be reciprocal. Implemented based on social learning theory (24), social skills training involves cognitive processes during modeling of desirable behaviors. Deficit in neurocognition has been found to be a significant barrier to the benefits from social skills training (25). Also, a meta-analysis has found that cognitive remediation training could only account for about 33% of improvement in functional outcomes (26). The benefit of cognitive remediation should be translated to behavioral aspects, in order to improve psychosocial functioning (27).

Lastly, although the impairments in neurocognition and social cognition were both core features of schizophrenia, they did not influence psychosocial functioning independently. Instead, social cognition significantly mediated the effect of neurocognition on functional outcomes, and this mediation effect accounted for 25% of variances in the outcomes (28). Computer-assisted neurocognitive training was found to result in improved empathy, in addition to several other domains of neurocognition (29). This suggested that the benefits of neurocognitive training on psychosocial functioning can be consolidated with augmentation of social cognition training. Similarly, given the linkage between metacognition and social cognition (16), it is also plausible to boost cognitive remediation by combining metacognition training with social cognition training.

Therefore, it is plausible to significantly enhance psychosocial functioning of patients with SSDs when they receive psychoeducation, motivational interviewing, various types of cognitive remediation training, or social skills training in combination. Specifically, the effective interventions may include any combinations of at least two of the above interventions (except for the combination of psychoeducation and motivational interviewing).

Given the accumulating randomized controlled trials (RCTs) testing the effects of the above-mentioned combined interventions in the past decade, it was timely to review and quantitatively synthesize their effects on psychosocial functioning, justifying the present systematic review and meta-analysis. However, the publication period, types of interventions, and participants to be covered should be specified.

According to a previous meta-analysis of RCTs on all types of psychosocial interventions for schizophrenia published in 2011 or earlier, the combined effect from at least two of the above interventions had not been tested (30). Another recently published network meta-analysis with RCTs on any type of psychosocial intervention for schizophrenia did not set limit in publication period of the studies to be included (31), and all the included studies on combined interventions were published after 2011. Hence, it is practical to review and synthesize RCTs published since 2011.

Cognitive behavioral therapy (CBT) is another evidence-based psychosocial intervention for patients with SSDs. CBT is delivered through individualized talks with therapists, and patients learn to cope with their psychotic symptoms by challenging their perceptions and beliefs and developing alternative interpretations for adaptative purposes (2). Hence, CBT addresses positive symptoms of schizophrenia more directly, compared with the psychosocial interventions mentioned above. Given the difference in primary treatment goals, and the recent meta-analysis on CBT and combined interventions that include CBT for psychosocial functioning (32), CBT is not included in the present systematic review and meta-analysis.

An emphasis on early psychosocial treatment for patients with first-episode psychosis (FEP) has been emerging. Studies have found that up to 96% of first-episode schizophrenia patients can reach clinical remission in a year of medical treatment (33), whereas an increased risk of persistent symptoms has been found in association with subsequent relapses of the disorder (34). FEP patients are expected to be benefited more from psychosocial interventions, compared with non-FEP patients. Various multicomponent interventions have been developed and carried out for FEP patients around the world, and these interventions generally include four to six psychosocial components, such as psychoeducation, CBT, family therapy, vocational support, and crisis management; the pooled effect of these multicomponent interventions on psychosocial functioning, compared with treatment-as-usual, was found to be significant (35). However, the synthesized effects of combined interventions with at least two approaches of psychoeducation, motivational interviewing, neurocognitive training, social cognition training, and social skills training remained unknown. It is meaningful to include FEP patients in the criteria of participants of the present systematic review and meta-analysis to explore the differences in treatment effects on psychosocial functioning between FEP and non-FEP patients.

In summary, the psychosocial approaches of psychoeducation, motivational interviewing, neurocognitive training, social cognition training, and social skills training have the potential to significantly enhance the psychosocial functioning of patients with SSDs when delivered in combination. However, it is imperative to synthesize and estimate their combined effectiveness. Therefore, the present systematic review and meta-analysis aimed to synthesize the effectiveness of combined interventions with at least two these five selected psychosocial approaches for psychosocial functioning of patients with SSDs, relative to all types of controls. Psychosocial functioning was identified as the primary outcome, whereas symptom severity, rehospitalization rate, quality of life, cognition outcomes, and employment outcomes were included as secondary outcomes. The present study also explored (i) the pooled relative effectiveness of all the combined interventions on psychosocial functioning, compared with stand-alone interventions/interventions with one less component and (ii) whether the effectiveness of combined interventions on psychosocial functioning would differ between FEP patients and non-FEP patients.

## Methodology

The present study followed the guidelines of Preferred Reporting Items for Systematic Reviews and Meta-Analyses (PRISMA).

### Search strategy

Full-text articles in the databases of PubMed, CINAHL Complete, Embase, and PsycINFO were searched from 1 January 2011 to 30 June 2021, using searches for the following terms appearing in titles and/or abstracts: (psychoeducation OR motivational interviewing OR neurocognitive training OR cognitive remediation training OR social skills training) AND (psychosis OR psychotic OR schizophrenia OR schizoaffective OR delusional). After searches had been conducted with those terms, filters were applied to identify articles that reported RCTs and were published from 2011 through 30 June 2021.

### Inclusion and exclusion criteria

All the searched results were imported to Endnote X9 to remove duplicates, and they were screened by two independent reviewers using the following criteria. Studies were included if (i) they were RCTs; (ii) participants were diagnosed with SSDs; (iii) they tested interventions that included at least two of the following five trainings: psychoeducation, motivational interviewing, neurocognitive training, social cognition training, and social skills training; (iv) psychosocial functioning was assessed at least at baseline and post-intervention; (v) they were reported in English; and (vi) they were available in full-text. Studies were excluded if they were: (i) studies with participants at the prodromal stage of psychosis or at high risk of psychosis; (ii) studies testing interventions only for caregivers or family members of patients with SSDs; (iii) the additional/expected treatment components in intervention group, compared with control group, included trainings other than the five ones specified in the inclusion criteria; (iv) the intervention tested only included psychoeducation and motivational interviewing.

### Study selection and data extraction

Using the inclusion and exclusion criteria, two independent reviewers (EL and ZL) first screened all the collected titles and abstracts to include the relevant ones. Then, the full text articles about the studies considered by at least one reviewer to be relevant were downloaded and evaluated by the two reviewers separately, as another round of screening. Disagreement between the two reviewers was resolved through face-to-face discussion, and a final list of included studies was mutually agreed upon by both reviewers.

One reviewer extracted the data from all the included studies, and the other reviewer cross-checked those data. The following data were extracted for qualitative synthesis: study location, study setting, characteristics of participants [i.e., sample size, percentage of females, mean age and standard deviation (SD), percentage of diagnosis with different schizophrenia spectrum disorders, illness duration (mean and SD in years), and whether identified as FEP], intervention duration, treatment components for intervention group and control group, and findings about related outcomes. For meta-analysis, the mean and SD of the primary outcomes (i.e., symptoms severity and psychosocial functioning) and of the secondary outcomes (i.e., rate and length of hospital readmission, quality of life, cognitive outcome, or employment outcome) at post-intervention were extracted. If no such data were available, the reviewers searched for a relevant dataset shared online and contacted the authors of the articles requesting their data.

### Quality assessment

The risk of bias in the included studies was assessed by the same two independent reviewers, using the Cochrane Risk of Bias 2 Tool (36). The assessed domains included the randomization process, deviations from the intended interventions, missing outcome data, measurement of outcomes, and selection of the reported results. Disagreement was resolved by face-to-face discussion.

### Data synthesis and analysis

The extracted qualitative data were tabulated for synthesis. Specifically, the data about the characteristics of participants in each study were tabulated, and another table was constructed to synthesize and compare the intervention characteristics of each study included. For meta-analyses, if an outcome was measured by the same scale, mean difference (MD) was used; if different scales were used across the studies to measure the same outcome, standard mean difference (SMD) was adopted. Publication bias was assessed with Egger’s regression test (37), conducted with excel algorism of Meta-Essentials (38). Review Manager 5.4 (39) was used to test heterogeneity (τ^2^, χ^2^, and *I*^2^) and calculate overall effect size. Given the variation in treatment components and duration across the included studies, high heterogeneity was expected, and random effect model was adopted for meta-analysis (40). Synthesized qualitative data was be compared between subgroups of FEP and non-FEP subjects. If there were at least three studies to be identified in FEP and non-FEP groups, subgroup analysis would be performed with Review Manager 5.4.

## Results

### Study selection

**FIGURE 1:**
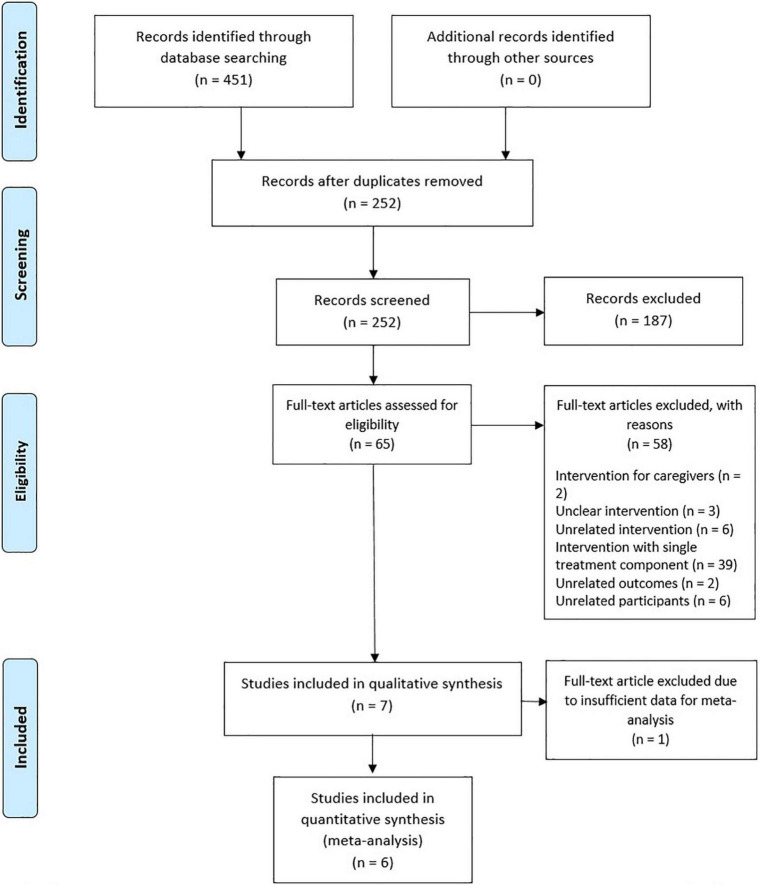
PRISMA flow diagram.

With the search strategy specified in Section “Search strategy,” 451 records were identified from the databases of PubMed, CINAHL Complete, Embase, and PsycINFO. After removing duplicates, a total of 252 records were screened by the two independent reviewers, based on titles and abstracts, and 187 records were excluded because they did not meet the inclusion criteria of the present study. Full-text articles of the remaining 65 records were assessed for eligibility, and that led to a further exclusion of 58 records. The primary reason for exclusion was if the intervention contained additional unrelated treatment components or the intervention with only one treatment component that was of interest in the present study, and other reasons were if caregivers were participants, or if there were unrelated interventions, outcomes, or participants. As a result, a remaining seven records were included for qualitative synthesis. For the meta-analysis, one study (41) was excluded for not reporting the mean and SD of the primary and secondary outcomes at post-intervention and instead reported the mean and SD of change score from baseline. One of the reviewers searched for a relevant dataset shared online and attempted to contact the authors for data, but that was in vain. The PRISMA flow diagram of the present study is shown in Figure 1.

### Study characteristics

The seven included studies were published in the period from 2012 through 2018 and involved 602 patients with schizophrenic spectrum disorders. The mean age of the patients ranged from 24.1 to 44.1 years. Only one study recruited patients with FEP (42). The studies had been conducted in varied locations, including two in Mexico (42, 43), one in Hong Kong SAR, China (44), two in the US (45, 46), one in Spain (47), and one in Japan (41). The study characteristics are summarized in Table 1.

**TABLE 1:** Summary of the characteristics of the included studies.

Study	Location	Setting	Sample size for analysis	Gender (% females)	Age [Mean (*SD*/range)]	Diagnosis	Illness duration [year; Mean (*SD*)]	FEP
Valencia et al. (42)	Mexico	Outpatient	I: 39 C: 34	I: 23.1% C: 26.5%	I: 24.5 (3.0) C: 24.1 (3.2)	100% with schizophrenia-spectrum disorders (% in subcategories unspecified)	Unspecified	Yes
Valencia et al. (43)	Mexico	Outpatient	I: 68 C: 51	I: 26.5% C: 23.5%	I: 29.5 (6.8) C: 26.4 (4.0)	100% with schizophrenia-spectrum disorders (% in subcategories unspecified)	I: 8.2 (5.3) C: 8.3 (6.5)	No
Au et al. (44)	Hong Kong SAR	Outpatient	I: 45 C: 45	I: 37.8% C: 35.6%	I: 35.38 (9.2) C: 36.89 (9.4)	-I: Schizophrenia (64.4%); schizoaffective disorder (35.6%) -C: Schizophrenia (51.1%); schizoaffective disorder (48.9%)	I: 11.33 (8.9) C: 11.08 (6.6)	No
Fisher et al. (45)	The US	Outpatient	I: 57 C: 54	I: 22.8% C: 35.2%	I: 44.08 (13.1) C: 42.37 (12.7)	-I: Schizophrenia (68.4%); schizoaffective disorder (29.8%); psychosis NOS (1.8%) -C: Schizophrenia (57.4%); schizoaffective disorder (40.7%); psychosis NOS (1.9%)	Unspecified	No
Inchausti et al. (47)	Spain	Outpatient	I: 36 C: 33	I: 44% C: 46%	I: 38.08 (12.1) C: 37.30 (13.0)	-I: Schizophrenia (50%); schizoaffective disorder (33%); delusional disorder (17%) -C: Schizophrenia (61%); schizoaffective disorder (27%); delusional disorder (12%)	I: 13.4 (8.9) C: 10.1 (7.9)	No
Lindenmayer et al. (46)	The US	Inpatient and Outpatient	I: 39 C: 39	I: 25.6% C: 30.8%	I: 41.0 (12.1) C: 42.7 (11.2)	-I: Schizophrenia (79.5%); schizoaffective disorder (2.1%) -C: Schizophrenia (82.1%); schizoaffective disorder (1.8%)	I: 14.34 (8.89) C: 15.22 (9.32)	No
Matsuda et al. (41)	Japan	Outpatient	I: 31 C: 31	I: 45.2% C: 41.9%	I: 36.4 (8.5) C: 37.8 (9.1)	100% with schizophrenia-spectrum disorders (% in subcategories unspecified)	Unspecified	No

C, control group; FEP, first-episode psychosis; I, intervention group; psychosis NOS, psychosis not otherwise specified; SD, standard deviation.

Regarding the interventions, treatment duration varied widely, ranging from 12 weeks to 1 year. All the included studies had two components of the following treatments for their intervention groups: psychoeducation, neurocognitive training, social cognition training, metacognition training, and social skills training. Other treatment components, if any, included pharmacological treatment, individual placement and support (IPS), and compensatory/bridging training, and these components were equivalent between intervention and control groups. The present study did not specify any restrictions about control conditions, and the control groups received a variety of treatments: treatment as usual (TAU), pharmacological treatment, and/or psychotherapies delivered to intervention groups, with fewer treatment components. The assessment tools for psychosocial functioning, the primary outcome, were varied across the studies, and included the Global Assessment of Functioning Scale (GAF) (48), the Personal and Social Performance Scale (49), the Social Functioning Scale (50), the Social and Occupational Functioning Assessment Scale (51), and the Life Assessment Scale for Mentally Ill (52). Summaries of the interventions, controls, and outcomes are reported in Table 2.

**TABLE 2:** Summary of the interventions, controls, and outcomes of the included studies.

Study	Intervention duration	Details of relevant treatment component(s)	Other treatment components	Controls	Relevant outcomes	Results
Valencia et al. (42)	1 year	**(1) PE** -For both patients and family –14 sessions (Session length unspecified) -Group training (Group size unspecified) -Content: (1) illness and management (10 sessions); (2) problem solving and communication (4 sessions) -Delivered by 2 family therapists **(2) SST** -For patients -Weekly sessions (no more than 75 min) over one year -Group training (6 patients per group) -Content: Medication, symptoms, skills to handle social and family relations -Delivered by 2 therapists	**Pharmacological treatment** -Prescription of antipsychotic medication and monthly consultation by 2 clinical psychiatrists	**Pharmacological treatment** -Prescription of antipsychotic medication and monthly consultation by 2 clinical psychiatrists	(1) Psychosocial functioning (GAF) (2) Symptoms severity (PANSS) (3) Re-hospitalization rate	**At post-intervention** 1. Significant improvement in psychological functioning found in intervention group only. (2) Significant improvement in symptomatology; greater improvement in negative symptoms, general psychopathology, and overall score in intervention group. (3) Lower rehospitalization rates in intervention group than control group
Valencia et al. (43)	6 months	**(1) PE** -For both patients and family –11 sessions (Session length unspecified) -Group training (Group size unspecified) -Content: (1) illness and management (8 sessions); (2) problem solving and communication (3 sessions) -Delivered by 2 family therapists **(2) SST** -For patients -Weekly sessions (no more than 60 min), 24 sessions over 6 months -Group training (group size unspecified) -Content: Medication, symptoms, skills to handle social and family relations -Delivered by 2 Ph.D. clinical psychologists	**Pharmacological treatment** -Prescription of antipsychotic medication and monthly consultation by 2 clinical psychiatrists	**Pharmacological treatment** -Prescription of antipsychotic medication and monthly consultation by 2 clinical psychiatrists	(1) Psychosocial functioning (GAF) (2) Symptoms severity (PANSS)	**At post-intervention** (1) No significant group differences in changes in psychosocial functioning. But only the intervention group showed significant improvement in psychosocial functioning overtime. (2) No significant group differences in changes in symptoms severity.
Au et al. (44)	3 months	**(1) NCT** -For patients -Maximum of 72 h, with 3 2-h sessions per week -Individual training -Computerized cognitive exercise -Therapists unspecified **(2) SST** -For patients –1.5 to 2.0-h session per week, 10 sessions/weeks -Group training (group size unspecified) -Content: Work-related social skills -Therapists unspecified	**IPS** -Six out of seven core features of the IPS were incorporated with the exception of the rapid job search.	**(1) IPS** Six out of seven core features of the IPS were incorporated except for the rapid job search. **(2) SST** Same as intervention group **(3)** A **TV watching** session was delivered	(1) Psychosocial functioning (GAF) (2) Symptoms severity (BPRS) (3) Cognitive function (4) Vocational status	**At post-intervention** (1) Both intervention and control groups showed improvement in psychosocial functioning; no significant group difference. (2) Both intervention and control groups showed improvement in symptoms severity; no significant group difference. (3) No significant group differences in cognitive function except for visual learning; intervention group had significantly higher score in visual learning. (4) No significant group differences in vocational status **At 11-month follow-up** (1) Both intervention and control groups showed improvement in psychosocial functioning; but control group had significantly higher score. (2) Both intervention and control groups showed improvement in symptoms severity; no significant group difference.
						(3) No significant group differences in cognitive function except for learning; intervention group had significantly higher score in learning. (4) Both groups showed significant improvement in job tenure. But no significant group difference in vocational status.
Fisher et al. (45)	14 weeks	**(1) NCT** -For Patients -Total 50 h, with a 1-h session per day, 5 sessions per week -Individual training -Content: computerized general auditory and visual exercise -Therapists unspecified **(2) SCT** -For patients -Total 20 hours, with a 1-h session per day, 5 sessions per week -Individual training -Content: computerized auditory and visual exercise about social cognition -Therapists unspecified	NA	**NCT** - Total 70 h, with a 1-h session per day, 5 sessions per week - Individual training - Content: computerized general auditory (40 h) and visual (30 h) exercise	(1) Psychosocial functioning (Social Functioning Scale) (2) Symptoms severity (PANSS) (3) Quality of life (4) Cognitive function	**At post-intervention** (1) No significant group differences in the changes of psychosocial functioning (2) No significant group differences in the changes of symptoms severity (3) No significant group differences in the changes of quality of life (4) Intervention group showed significantly greater improvement in social cognition and reward processing than control group did.
Inchausti et al. (47)	16 weeks	**A hybrid intervention** -For patients -90-min session, 1 session per week for 16 weeks -Group training with 8–12 patients per group -Delivered by clinical psychologists and occupational therapists **-With following components:** **(1) MCT** -Content: Training modules of self-reflexivity and understanding others **(2) SST** -Content: Trainings of conversation skills, assertive skills, and conflict management skills	NA	**Conventional SST** -For patients -90-min session, 1 session per week for 16 weeks -Group training with 8–12 patients per group -Content: Trainings of conversation skills, assertive skills, and conflict management skills -Delivered by clinical psychologists and occupational therapists	(1) Psychosocial functioning (Social and Occupational Functioning Assessment Scale) (2) Symptoms severity (PANSS) (23) Cognitive function	**At post-intervention** (1) Intervention group showed significantly greater improvement in psychosocial functioning than control group. (2) No significant group differences in the changes of symptoms severity (3) Intervention group showed significantly greater improvement in metacognitive function than control group. **At 6-month follow-up** (1) Intervention group showed significantly greater improvement in psychosocial functioning than control group. (2) No significant group differences in the changes of symptoms severity (3) Intervention group showed significantly greater improvement in metacognitive function than control group.
Lindenmayer et al. (46)	12 weeks	**(1) NCT** -For patients -2 h weekly for 12 weeks -Group training (8–10/group) -Content: computerized cognitive training with software COGPACK or Brain Fitness -Facilitated by two cognitive therapists who were Masters’ level and/or Ph.D. level psychologists	**Bridging group training** -1 h weekly for 12 weeks -Application of cognitive skills in daily function	**(1) NCT** Same as I **(2) Writing group** 1 h weekly for 12 weeks **(3) Bridging group training** Same as I	(1) Psychosocial functioning (Personal and Social Performance Scale) (2) Symptoms severity (PANSS) (3) Cognitive function	**At post-intervention** (1) No significant group differences in the changes of psychosocial functioning (2) No significant group differences in the changes of symptoms severity (3) Intervention group showed greater improvement in visual learning, working memory, reasoning, and social cognition than control group did.
		**(2) SCT** -For patients -1 h weekly for 12 weeks -Group training (8–10/group) -Content: computerized social cognition training with software MRIGE -Facilitated by two cognitive therapists who were Masters’ level and/or Ph.D. level psychologists				
Matsuda et al. (41)	12 weeks	**(1) NCT** -For patients -60 min session, 2 sessions/week, for 12 weeks -Training format unspecified - Content: computerized cognitive training with software JCORES -Therapists guided the training process **(2) SST** -For patients -1 session/week for 12 weeks -Group training -Content: Training to facilitate daily function and work performance -Therapists guided the training process	**TAU** Standard treatment in an outpatient unit	**TAU** Standard treatment in an outpatient unit	(1) Psychosocial functioning (Life Assessment Scale for Mentally Ill) (2) Symptoms severity (PANSS) (3) Cognitive function	**At post-intervention** (1) No significant group differences in the changes of psychosocial functioning (2) Intervention group showed greater improvement in general psychopathology than control group, but not in positive or negative symptoms. (3) Intervention group showed greater improvement in verbal memory and cognitive function in general.

BPRS, brief psychiatric rating scale; GAF, global assessment of functioning scale; IPS, individual placement and support; NA, not applicable; NCT, neurocognitive training; PANSS, positive and negative syndrome scale; PE, psychoeducation; SCT, social cognition training; MCT, metacognition training; SST, social skills training; TAU, treatment as usual.

### Risk of bias in the studies

**FIGURE 2:**
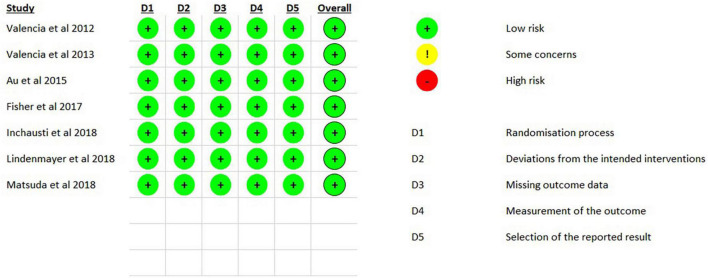
Risk of bias.

Figure 2 summarizes the risk of bias in the included studies. In general, the studies were of good quality and had a low risk of bias.

### Results of individual studies

The findings about the related outcomes of each study are summarized in Table 2. All the seven included studies evaluated treatment effects at post-intervention, and only two studies (44, 47) included follow-up assessments to test maintenance effect of combined psychosocial interventions 6 months later or longer.

#### Primary outcome: Psychosocial functioning

The intervention groups of two studies (42, 47) showed significantly greater improvement than the control groups did at post-intervention. Although intervention groups of the remaining studies (41, 43–46) did not improve significantly more than the control groups did, a trend that favored the intervention groups was observed in three studies (41, 43, 45). Improvement in both intervention and control groups was reported in the remaining two studies (44, 46).

Au et al. (44) reported continuing improvement in psychosocial functioning in both intervention and control groups at 7-month and 11-month follow-up; however, control group showed significantly higher score in psychosocial functioning at 11-month follow-up. As for Inchausti et al. study (47), the significant effect on psychosocial functioning in intervention group maintained at 6-month follow-up.

In addition, as the only one with patients with FEP, Valencia et al. (42) reported statistically significant treatment effects on psychosocial functioning. Comparatively, a trend that favored intervention groups to have more improvement in psychosocial functioning can be found in the remaining studies on non-FEP patients.

#### Secondary outcomes

Two studies (41, 42) reported a significantly greater score reduction of symptom severity in the intervention group, compared with the control group. A significant Group*Time interaction effect was found for negative symptoms, general psychopathology, and overall symptom severity score in Valencia and colleagues’ study (42), whereas Matsuda et al. (41) reported significantly greater improvement only in general psychopathology in the intervention group than control group. The remaining five studies reported insignificant group differences in the changes in symptoms severity (43–47), and decreasing trends of symptom severity scores in both the intervention group and the control group were observed. As for the two studies evaluating maintenance effect, neither of them found significant effect on symptom severities at follow-ups of 6 months or longer (44, 47).

The rate of hospitalization was assessed in one study (42), and it found a greater reduction of hospitalization rate in the intervention group than in the control group. Quality of life was reported in one study (45), but it reported insignificant difference between the intervention group and control group in the patients’ change in quality of life. Fisher et al. (45) and Lindenmayer et al. (46) reported significantly greater enhancement in social cognition in the intervention groups than in the control groups. Inchausti et al. (47) reported significantly more improvement in metacognition in intervention group, compared with control group, at both post-intervention and 6-month follow-up. Various domains of neurocognition were assessed in four studies (41, 44–46), and significantly more improvement in intervention group, compared with control group, in at least one domain were reported in three studies (41, 44, 46). In addition, Au and colleagues (44) reported significantly better learning function in intervention group at 11-month follow-up. Employment outcomes were assessed by Au et al. (44), and they reported insignificant group differences in employment duration after intervention. However, Au et al. (44) did observe a time effect at 7-month and 11-month follow-ups that the patients in both intervention and control groups showed significantly better status of job tenure.

### Meta-analyses of primary and secondary outcomes

To test for publication bias, Egger’s regression test was performed for all the primary and secondary outcomes that were eligible for meta-analyses. The two-tailed *p*-values of Egger’s regression test for all outcomes were higher than.05, indicating low risk of publication bias.

**FIGURE 3:**
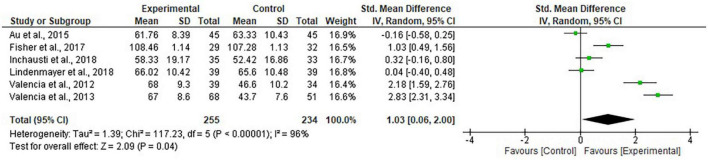
Forest plot of psychosocial functioning.

The forest plot of meta-analysis on psychosocial functioning is shown in Figure 3. Sensitivity analyses were performed on psychosocial functioning and summarized in Table 3. The results of meta-analyses on all the eligible secondary outcomes (mainly outcomes related to symptom severity and cognitive function) were reported in Table 4.

**TABLE 3:** Meta-analyses of the effects at post-intervention on psychosocial functioning (primary outcome).

Included studies	Sample (*N*)	Total mean difference (95% CI)	*Z*	*p*	Heterogeneity
All the included studies:**** Valencia et al. (42, 43)**** Au et al. (44)**** Fisher et al. (45)**** Lindenmayer et al. (46)**** Inchausti et al. (47)	I: 255 C: 234	SMD = 1.03 [0.06, 2.00]	2.09	0.04	τ^2^ = 1.39 χ^2^ = 117.23, df = 5, *p* < 0.001 *I*^2^ = 96%
Studies working with non-FEP participants:**** Valencia et al. (43) Au et al. (44)**** Fisher et al. (45)**** Lindenmayer et al. (46) Inchausti et al. (47)****	I: 216 C: 200	SMD = 0.80 [–0.23, 1.83]	1.53	0.13	τ^2^ = 1.32 χ^2^ = 94.47, df = 4, *p* < 0.001 *I*^2^ = 96%
Studies testing the effects relative to control conditions with one less intervention component:**** Au et al. (44)**** Fisher et al. (45)**** Lindenmayer et al. (46) Inchausti et al. (47)	I: 148 C: 149	SMD = 0.29 [–0.20, 0.77]	1.16	0.24	τ^2^ = 0.18 χ^2^ = 12.80, df = 3, *p* = 0.005 *I*^2^ = 77%

FEP, first-episode psychosis; PE, psychoeducation; SST, social skills training; TAU, treatment as usual; I, intervention group; C, control group; SMD, standard mean difference; MD, mean difference.

**TABLE 4:** Meta-analyses of the effects at post-intervention on secondary outcomes.

Outcome	Included studies	Sample (*N*)	Total mean difference (95% CI)	*Z*	*p*	Heterogeneity
Positive symptoms	Valencia et al. (42, 43) Fisher et al. (45) Lindenmayer et al. (46) Inchausti et al. (47)	I: 210 C: 189	*MD* = –0.28 [–1.82, 1.26]	0.36	0.72	τ^2^ = 2.12 χ^2^ = 18.33, df = 4, *p* < 0.001 *I*^2^ = 78%
Negative symptoms	Valencia et al. (42, 43) Fisher et al. (45) Lindenmayer et al. (46) Inchausti et al. (47)	I: 210 C: 189	*MD* = –1.26 [–3.68, 1.16]	1.02	0.31	τ^2^ = 6.70 χ^2^ = 51.24, df = 4, *p* < 0.001 *I*^2^ = 92%
General psychopathology	Valencia et al. (42) Fisher et al. (45) Inchausti et al. (47)	I: 103 C: 99	*MD* = –1.66 [–7.33, 4.00]	0.58	0.57	τ^2^ = 22.29 χ^2^ = 25.27, df = 2, *p* < 0.001 *I*^2^ = 92%
Overall score of symptom severity	Valencia et al. (42) Au et al. (44) Fisher et al. (45) Lindenmayer et al. (46) Inchausti et al. (47)	I: 187 C: 183	SMD = 0.22 [–0.65, 1.10]	0.51	0.61	τ^2^ = 0.92 χ^2^ = 64.64, df = 4, *p* < 0.001 *I*^2^ = 94%
Speed of processing	Au et al. (44) Fisher et al. (45) Lindenmayer et al. (46)	I: 113 C: 116	*MD* = –2.49 [–7.28, 2.30]	1.02	0.31	τ^2^ = 9.98 χ^2^ = 4.41, df = 2, *p* = 0.11 *I*^2^ = 55%
Visual learning	Au et al. (44) Fisher et al. (45) Lindenmayer et al. (46)	I: 113 C: 116	*MD* = 1.34 [–1.13, 3.82]	1.06	0.29	τ^2^ = 2.67 χ^2^ = 4.49, df = 2, *p* = 0.11 *I*^2^ = 55%
Reasoning and problem solving	Au et al. (44) Fisher et al. (45) Lindenmayer et al. (46)	I: 113 C: 116	MD = –0.42 [–2.48, 1.65]	0.39	0.69	τ^2^ = 1.73 χ^2^ = 3.81, df = 2, *p* = 0.15 *I*^2^ = 47%
Attention/vigilance	Au et al. (44) Fisher et al. (45) Lindenmayer et al. (46)	I: 113 C: 116	*MD* = 0.57 [–4.31, 5.44]	0.23	0.82	τ^2^ = 14.38 χ^2^ = 9.83, df = 2, *p* = 0.007 *I*^2^ = 8-%

I, intervention group; C, control group; SMD, standard mean difference; MD, mean difference.

#### Primary outcomes

All the six studies assessed psychosocial functioning, using various assessment tools, and their pooled effect was significant (SMD = 1.03, 95% CI [0.06, 2.00], *Z* = 2.09, *p* = 0.04, *I*^2^ = 96%; Figure 3). Since there was only one of the included studies recruiting patients with FEP (42), it was not statistically sufficient to compare the pooled effects between FEP and non-FEP subgroups. Sensitivity analysis was performed to test the effects on psychosocial functioning within the non-FEP subgroup. However, the effects become insignificant for the non-FEP subgroup (SMD = 0.80, 95% CI [–0.23, 1.83], *Z* = 1.53, *p* = 0.13, *I*^2^ = 96%; see Table 3). Another sensitivity analysis was conducted, excluding two studies (42, 43), to obtain the pooled effect with studies testing the relative effects of combined interventions compared with single-component interventions/interventions with one less component; the additional components in intervention groups included neurocognition training, social cognition training, or social skills training. However, their pooled effect was not significant (SMD = 0.80, 95% CI [–0.23, 1.83], *Z* = 1.53, *p* = 0.13, *I*^2^ = 96%; Table 3). All the six studies incorporated different combinations of treatment elements for intervention groups and control groups and/or recruited different types of patients (FEP vs. non-FEP). Hence, no subgroup synthesis within these studies could be performed.

#### Secondary outcomes

Meta-analyses were conducted for positive symptoms, negative symptoms, general psychopathology, overall score of symptom severity, and some specific domains of neurocognition (such as speed of processing, visual learning, reasoning and problem solving, and attention/vigilance), and the results are summarized in Table 4. No significant pooled effects were found in any of the secondary outcomes. The data extracted for these analyses are reported in Supplementary File.

## Discussion

Based on seven good-quality studies with 602 patients with SSDs, the present systematic review and meta-analysis synthesized the effects of combined interventions that include two of the following psychosocial treatment elements: psychoeducation, neurocognitive training, social cognition training, metacognition training, and social skills training, compared with all types of control conditions. For the primary outcome, psychosocial functioning, five out of the seven studies reported significant effects or positive trend that favored intervention group at post-intervention. Long-term effects of the combined interventions on psychosocial functioning remained inconclusive given mixed findings from only two of the included studies. Meta-analysis with the six eligible studies showed a significant pooled effect on psychosocial functioning at post-intervention, but the effect became insignificant in the following analyses: (i) quantitative synthesis of the five studies working with non-FEP patients, and (ii) quantitative synthesis of the four studies testing the relative effects of the combined interventions compared with stand-alone interventions/interventions with one less component.

As for the secondary outcomes, the evidence on symptom severity, as assessed in all the seven studies, generally not supported the efficacy of the combined interventions. Significant improvement in neurocognition was reported in majority of the studies adopting neurocognitive training as one component of the combined intervention. Evident enhancement of social cognition was found in two studies. There was also evidence in different single studies about the treatment-related effects on hospitalization rate, metacognition, and employment. Meta-analysis revealed that the combined intervention did not outperform control conditions to reduce symptoms severity or enhance neurocognition.

Although motivational interviewing was one of the psychosocial interventions to be reviewed, no included study incorporated this intervention. It was adopted as one of the treatment components in some of the excluded studies, which involve additional psychosocial interventions, such as CBT, family therapy, and individualized occupational therapy. Hence, the research question regarding the efficacy of combined interventions that include motivational interviewing for the enhancement of psychosocial functioning could not be answered in the present systematic review and meta-analysis. The secondary aim to explore the differences in efficacy in FEP and non-FEP patients could not be fully addressed, either, since only one of the included studies worked with FEP patients. While larger effect sizes of improvement in psychosocial functioning and symptom severity were observed in Valencia and colleagues’ study (42), it would be premature to conclude any differences in treatment effects for FEP and non-FEP patients.

All the included studies adopted the combinations of psychosocial approaches as reviewed in the Introduction, namely, (i) psychoeducation + social skills training, (ii) any type of cognitive remediation training + social skills training, and (iii) the combination of different types of cognitive remediation training. Hence, the significant pooled effect on psychosocial functioning with large effect size, as found in the present study, supported the speculation that these psychosocial interventions can augment each other given the close associations and interactions among motivation, cognition, and behavior as enhanced by the interventions. For example, the evident improvement in psychosocial functioning in the intervention groups of Valencia and colleagues’ studies (42, 43) might not be merely attributed to social skills training; psychoeducation also played a significant role given lower attrition rate and higher medication compliance in the intervention groups. The substantial improvement in psychosocial functioning in the intervention group of Inchausti et al. study (47) reflected the important role of metacognition in social interaction. Moreover, although without significant effects on psychosocial functioning, Fisher et al. (45) and Lindermayer et al. (46) have demonstrated that combined interventions with neurocognition as one of the components can results in improvement in social cognition, and vice versa; which in turn, will be translated to improved psychosocial functioning in longer term. In summary, the significant effect on psychosocial functioning informs the strategy to deliver more than one type of psychosocial intervention for patients with SSDs within the same treatment period to improve their psychosocial functioning, and optimal treatment benefits would be expected if patients can receive all three categories of interventions, namely psychoeducation, cognitive remediation training, and social skills training.

However, it is important to note the insignificant effects on psychosocial functioning as resulted from the meta-analyses of a subgroup of the five studies with non-FEP patients and another subgroup of the four studies testing relative effects of combined interventions compared with stand-alone interventions/interventions with one less component. The participants of the latter subgroup were also all non-FEP patients. One explanation about the insignificant results may be the limited number of studies, making it underpowered to detect a significant pooled effect with small effect size. As FEP patients are expected to benefit more from treatments than non-FEP patients, smaller treatment-related improvement in psychosocial functioning would be plausible within the non-FEP subgroup. The effect size of the effects of the combined interventions, relative to stand-alone interventions/interventions with one less component, are also likely to be smaller than the ones from a comparison with TAU. Such drops in standard mean differences can be observed in Table 3. The other explanation could be the limited acute effect of the combined interventions on psychosocial functioning, especially those incorporated one or two components of cognitive remediation training (44–47). Fisher et al. (45), Lindermayer et al. (46), and Inchausti et al. (47) all proposed that it would take some time for cognitive gains to be translated to enhancement in psychosocial functioning, and such benefits might not be detectable at post-intervention.

Given the limited number of included studies eligible for meta-analyses and the heterogeneity in intervention combinations, control conditions, and participants, it was not feasible to further analyze the pooled effect in any specific types of combined intervention. The present findings also cannot be compared quantitatively with recent meta-analyses on the efficacy of psychoeducation, cognitive remediation training, or social skills training as stand-alone intervention for psychosocial functioning of patients with SSDs. According to Xia and colleagues (6), the effect of psychoeducation on psychosocial functioning, compared to TAU, was unclear due to the limited number of eligible studies and their mixed findings. Similar inconclusive effects were reported in Almerie et al. systematic review and meta-analysis on social skill training (53), given the general low research quality in the included studies. A very recent meta-analysis by Vita et al. (54) has found that cognitive remediation training had a significant and small effect on general functioning at post-intervention, compared to all types of control conditions. All the included studies that adopted cognitive remediation training as one of the intervention components all happened to test the effect of the combined interventions relative to stand-alone interventions/interventions with one less component (44–47), and this was not directly comparable to the effect found by Vita et al. (54). In summary, it remains unknown whether specific combinations of intervention as reviewed in the present study can elicit stronger effect than stand-alone intervention. We are conducting another systematic review and meta-analysis to synthesize the effects of psychoeducation, different types of cognitive remediation training, and social skills training as stand-alone intervention on psychosocial functioning, compared to all types of control conditions in general, and the findings of this review can inform the different efficacies of combined versus stand-alone intervention.

Although the efficacy of specific combination of interventions cannot be derived from the present review, qualitative synthesis of the findings may suggest the importance of *how* different interventions should be combined. According to two studies with significant improvements in psychosocial functioning (42, 47), the treatments for intervention groups were not mere additions of two different psychosocial interventions (as reported in the other included studies); instead, connections between two interventions were strong. Specifically, the psychoeducation sessions in Valencia et al. study (42) covered issues of problem solving and communication, and relevant skills were learnt and practiced during social skills training sessions. A hybrid mode of different intervention components was adopted in Inchausti et al. study (47), and each training session for the intervention group consisted of metacognition training in the first half and training of different areas of social skills with metacognition applied in the second half.

It was not surprising to have insignificant pooled effects on symptom severity since the primary treatment goals of the reviewed psychosocial interventions are not about the reduction of positive, negative, or general symptoms of schizophrenia spectrum disorders. Previous studies had found a significantly stronger treatment effect from psychosocial interventions combined with medication, compared with psychosocial intervention alone or medication alone (55, 56). This is confirmed in three of the included studies (41–43), which all compared combined psychosocial intervention + medication with medication alone. However, the remaining studies tested the effect of combined interventions relative to stand-alone interventions/interventions with one less component, and medication dosage was either equivalent between groups or unclear. This suggested that the addition of psychosocial interventions of psychoeducation, cognitive remediation training, social skills training was not sufficient to reduce symptom severity.

As shown in Table 4, the pooled insignificant effects on neurocognition were all derived from the findings of the same three studies (44–46). Neurocognition training was provided to both intervention and control groups in Fisher et al. study (45) and Lindermayer et al. study (46), and neurocognitive functions of both groups were improved. This may explain the insignificant pooled effect. However, Au et al. (44) also reported similar increases in neurocognition scores in both groups, suggesting that neurocognition training, in addition to social skills training and individual placement and support, could not produce further improvement in neurocognitive functions.

Three limitations of the present systematic review and meta-analysis should be noted. The first limitation was the failure to include enough studies with motivational interviewing as one component of combined intervention or enough studies with FEP patients as participants. Research questions for future relevant systematic reviews should be revised to ensure feasibility. Scoping reviews on (i) the application of motivational interviewing in patients with SSDs and (ii) the current status of psychosocial interventions for FEP patients may be important future directions. Also, future RCTs should test the efficacy of combined interventions with motivational interviewing + cognitive remediation and/or social skills training to improve psychosocial functioning of patients with SSDs. Although there have been many RCTs on multi-component and integrated treatment for FEP patients, it would also be meaningful to examine the efficacy of different pairs of psychosocial interventions provided in combination to identify core elements in the integrated treatment. The second limitation was the exclusion of Matsuda et al. study (41) from meta-analysis given its insufficient data. More improvement in intervention group than control group was reported, but such difference was not significant (41). It would be difficult to predict how the overall effects would be changed if this study was included. The last limitation was about the limited number of studies testing the maintenance effect of combined psychosocial interventions on psychosocial functioning. While it may take time for treatment-related enhancement in motivation, cognition, and/or behavior to benefit psychosocial functioning, this cannot be clear based on the present systematic review and meta-analysis. Future RCTs on the combined psychosocial interventions should employ more follow-up assessments on psychosocial functioning.

## Conclusion

The present systematic review and meta-analysis found significant effects from combinations of psychoeducation, cognitive remediation training, and social skills training on the psychosocial functioning of patients with SSDs, compared with all control conditions. This supported the provision of more than one psychosocial intervention from the above three types to facilitate patients’ daily functioning. It appeared that the benefits of such combined interventions could be boosted if they were delivered in a connected and hybrid mode. However, more studies testing the effects of different specific combinations of interventions should be conducted, especially those in comparison with stand-alone interventions and working with FEP patients. It turned out to be infeasible to synthesize the effects from combined interventions that include motivational interviewing or to compare the effect size between FEP and non-FEP patients. These issues may be addressed in future scoping reviews.

## Data availability statement

The original contributions presented in this study are included in the article/Supplementary material, further inquiries can be directed to the corresponding author.

## Author contributions

EL contributed to research design, systematic search and screening, data extraction, synthesis and analysis, interpretation of research findings, and manuscript writing. AC contributed to research design, interpretation of research findings, and manuscript writing. HT contributed to research design, interpretation of research findings, and manuscript finalizing. JC, SL, AY, JL, WZ, MZ, and NM contributed to interpretation of research findings and manuscript finalizing. ZL contributed to systematic search, screening, data extraction, and data analysis. All authors contributed to the article and approved the submitted version.

## References

[1] Charlson FJ Ferrari AJ Santomauro DF Diminic S Stockings E Scott JG Global Epidemiology and burden of schizophrenia: findings From the global burden of disease study 2016. *Schizophr Bull.* (2018) 44:1195–203. 10.1093/schbul/sby058 29762765 PMC6192504

[2] Chien WT Yip ALK. Current approaches to treatments for schizophrenia spectrum disorders, part I: an overview and medical treatments. *Neuropsychiatr Dis Treat.* (2013) 9:1311–32. 10.2147/NDT.S37485 24049446 PMC3775702

[3] Leucht S Arbter D Engel RR Kissling W Davis JM. How effective are second-generation antipsychotic drugs? A meta-analysis of placebo-controlled trials. *Mol Psychiatry.* (2009) 14:429–47. 10.1038/sj.mp.4002136 18180760

[4] Tandon R Lenderking WR Weiss C Shalhoub H Barbosa CD Chen J The impact on functioning of second-generation antipsychotic medication side effects for patients with schizophrenia: a worldwide, cross-sectional, web-based survey. *Ann Gen Psychiatry.* (2020) 19:42. 10.1186/s12991-020-00292-5 32684942 PMC7359579

[5] Zubin J Spring B. Vulnerability: a new view of schizophrenia. *J Abnorm Psychol.* (1977) 86:103. 10.1037/0021-843X.86.2.103 858828

[6] Xia J Merinder LB Belgamwar MR. Psychoeducation for Schizophrenia. *Schizophr Bull.* (2011) 37:21–2. 10.1093/schbul/sbq138 21147896 PMC3004189

[7] Miller WR Rollnick S. *Motivational Interviewing: Preparing People For Change.* New York, NY: Guilford Press (2002).

[8] Hettema J Steele J Miller WR. Motivational interviewing. *Annu Rev Clin Psychol.* (2005) 1:91–111. 10.1146/annurev.clinpsy.1.102803.143833 17716083

[9] Fiszdon JM Kurtz MM Choi J Bell MD Martino S. Motivational interviewing to increase cognitive rehabilitation adherence in schizophrenia. *Schizophr Bull.* (2016) 42:327–34. 10.1093/schbul/sbv143 26420905 PMC4753608

[10] Prikke M Konings MJ Lei WU Begemann MJH Sommer IEC. The efficacy of computerized cognitive drill and practice training for patients with a schizophrenia-spectrum disorder: a meta-analysis. *Schizophr Res.* (2019) 204:368–74. 10.1016/j.schres.2018.07.034 30097278

[11] Tan BL Lee SA Lee J. Social cognitive interventions for people with schizophrenia: a systematic review. *Asian J Psychiatry.* (2018) 35:115–31. 10.1016/j.ajp.2016.06.013 27670776

[12] d’Arma A Isernia S Di Tella S Rovaris M Valle A Baglio F Social cognition training for enhancing affective and cognitive theory of mind in schizophrenia: a systematic review and a meta-analysis. *J Psychol.* (2021) 155:26–58. 10.1080/00223980.2020.1818671 33048659

[13] Horan WP Dolinsky M Lee J Kern RS Hellemann G Sugar CA Social cognitive skills training for psychosis with community-based training exercises: a randomized controlled trial. *Schizophr Bull.* (2018) 44:1254–66. 10.1093/schbul/sbx167 29300973 PMC6192506

[14] Granholm E Holden J Link PC McQuaid JR. Randomized clinical trial of cognitive behavioral social skills training for schizophrenia: improvement in functioning and experiential negative symptoms. *J Consul Clin Psychol.* (2014) 82:1173–85. 10.1037/a0037098 24911420 PMC4244255

[15] Flavell JH. Metacognition and cognitive monitoring: a new area of cognitive–developmental inquiry. *Am Psychol.* (1979) 34:906–11. 10.1037/0003-066X.34.10.906

[16] Kukla M Lysaker PH. Metacognition over time is related to neurocognition, social cognition, and intrapsychic foundations in psychosis. *Schizophr Res Cogn.* (2020) 19:100149. 10.1016/j.scog.2019.100149 31832339 PMC6889797

[17] Moritz S Thoering T Kühn S Willenborg B Westermann S Nagel M. Metacognition-augmented cognitive remediation training reduces jumping to conclusions and overconfidence but not neurocognitive deficits in psychosis. *Front Psychol.* (2015) 6:1048. 10.3389/fpsyg.2015.01048 26283990 PMC4522518

[18] Cella M Edwards C Swan S Elliot K Reeder C Wykes TJ. Exploring the effects of cognitive remediation on metacognition in people with schizophrenia. *J Exp Psychopathol.* (2019) 10:2043808719826846. 10.1177/2043808719826846

[19] Kopelowicz A Liberman RP Zarate R. Recent advances in social skills training for schizophrenia. *Schizophr Bull.* (2006) 32:S12–23. 10.1093/schbul/sbl023 16885207 PMC2632540

[20] Almerie MQ AlMarhi MO Jawoosh M Alsabbagh M Matar HE Maayan N Social skills programmes for schizophrenia. *Cochrane Database Syst Rev.* (2015) 2015:CD009006. 10.1002/14651858.CD009006.pub2 26059249 PMC7033904

[21] Deci EL Ryan RM. *Intrinsic Motivation And Self-Determination In Human Behavior.* Berlin: Springer Science & Business Media (2013).

[22] Choi J Medalia A. Intrinsic motivation and learning in a schizophrenia spectrum sample. *Schizophr Res.* (2010) 118:12–9. 10.1016/j.schres.2009.08.001 19716270 PMC2856796

[23] Najas-Garcia A Gomez-Benito J Huedo-Medina TB. The relationship of motivation and neurocognition with functionality in schizophrenia: a meta-analytic review. *Community Ment Health J.* (2018) 54:1019–49. 10.1007/s10597-018-0266-4 29605875

[24] Bandura A Walters RH. *Social Learning Theory.* Englewood Cliffs, NJ: Prentice Hall (1977).

[25] Kurtz MM. Neurocognition as a predictor of response to evidence-based psychosocial interventions in schizophrenia: what is the state of the evidence? *Clin Psychol Rev.* (2011) 31:663–72. 10.1016/j.cpr.2011.02.008 21482324 PMC3075917

[26] Fett A-KJ Viechtbauer W Penn DL van Os J Krabbendam L. The relationship between neurocognition and social cognition with functional outcomes in schizophrenia: a meta-analysis. *Neurosci Biobehav Rev.* (2011) 35:573–88. 10.1016/j.neubiorev.2010.07.001 20620163

[27] Bowie CR McGurk SR Mausbach B Patterson TL Harvey PD. Combined cognitive remediation and functional skills training for schizophrenia: effects on cognition, functional competence, and real-world behavior. *Am J Psychiatry.* (2012) 169:710–8. 10.1176/appi.ajp.2012.11091337 22581070

[28] Schmidt SJ Mueller DR Roder V. Social cognition as a mediator variable between neurocognition and functional outcome in schizophrenia: empirical review and new results by structural equation modeling. *Schizophr Bull.* (2011) 37(suppl. 2):S41–54. 10.1093/schbul/sbr079 21860046 PMC3160114

[29] Kurtz MM Mueser KT Thime WR Corbera S Wexler BE. Social skills training and computer-assisted cognitive remediation in schizophrenia. *Schizophr Res.* (2015) 162:35–41. 10.1016/j.schres.2015.01.020 25640526 PMC5146951

[30] De Silva MJ Cooper S Li HL Lund C Patel V. Effect of psychosocial interventions on social functioning in depression and schizophrenia: meta-analysis. *Br J Psychiatry.* (2013) 202:253–60. 10.1192/bjp.bp.112.118018 23549941 PMC3613719

[31] McGlanaghy E Turner D Davis GA Sharpe H Dougall N Morris P A network meta-analysis of psychological interventions for schizophrenia and psychosis: impact on symptoms. *Schizophr Res.* (2021) 228:447–59. 10.1016/j.schres.2020.12.036 33578368

[32] Laws KR Darlington N Kondel TK McKenna PJ Jauhar S. Cognitive Behavioural Therapy for schizophrenia-outcomes for functioning, distress and quality of life: a meta-analysis. *BMC Psychol.* (2018) 6:32. 10.1186/s40359-018-0243-2 30016999 PMC6050679

[33] Robinson DG Woerner MG Delman HM Kane JM. Pharmacological treatments for first-episode schizophrenia. *Schizophr Bull.* (2005) 31:705–22. 10.1093/schbul/sbi032 16006592

[34] Wiersma D Nienhuis FJ Slooff CJ Giel R. Natural course of schizophrenic disorders: a 15-year followup of a dutch incidence cohort. *Schizophr Bull.* (1998) 24:75–85. 10.1093/oxfordjournals.schbul.a033315 9502547

[35] Correll CU Galling B Pawar A Krivko A Bonetto C Ruggeri M Comparison of early intervention services vs treatment as usual for early-phase psychosis: a systematic review, meta-analysis, and meta-regression. *JAMA Psychiatry.* (2018) 75:555–65. 10.1001/jamapsychiatry.2018.0623 29800949 PMC6137532

[36] Sterne JAC Savovic J Page MJ Elbers RG Blencowe NS Boutron I RoB 2: a revised tool for assessing risk of bias in randomised trials. *BMJ-Br Med J.* (2019) 366:l4898. 10.1136/bmj.l4898 31462531

[37] Egger M Smith GD Schneider M Minder C. Bias in meta-analysis detected by a simple, graphical test. *BMJ-Br Med J.* (1997) 315:629–34. 10.1136/bmj.315.7109.629 9310563 PMC2127453

[38] Suurmond R van Rhee H Hak T. Introduction, comparison, and validation of Meta-Essentials: a free and simple tool for meta-analysis. *Res Synth Methods.* (2017) 8:537–53. 10.1002/jrsm.1260 28801932 PMC5725669

[39] The Cochrane Collaboration. *Review Manager (RevMan) Version 5.4.* (n.d.). Available online at: https://training.cochrane.org/online-learning/core-software-cochrane-reviews/revman (accessed Sept 1, 2021).

[40] Borenstein M Hedges LV Higgins JP Rothstein HR. A basic introduction to fixed-effect and random-effects models for meta-analysis. *Res Synth Methods.* (2010) 1:97–111. 10.1002/jrsm.12 26061376

[41] Matsuda Y Morimoto T Furukawa S Sato S Hatsuse N Iwata K Feasibility and effectiveness of a cognitive remediation programme with original computerised cognitive training and group intervention for schizophrenia: a multicentre randomised trial. *Neuropsychol Rehabil.* (2018) 28:387–97. 10.1080/09602011.2016.1181555 27150346

[42] Valencia M Juarez F Ortega H. Integrated treatment to achieve functional recovery for first-episode psychosis. *Schizophr Res Treat.* (2012) 2012:962371. 10.1155/2012/962371 22970366 PMC3420493

[43] Valencia M Fresan A Juárez F Escamilla R Saracco R. The beneficial effects of combining pharmacological and psychosocial treatment on remission and functional outcome in outpatients with schizophrenia. *J Psychiatr Res.* (2013) 47:1886–92. 10.1016/j.jpsychires.2013.09.006 24112947

[44] Au DWH Tsang HWH So WWY Bell MD Cheung V Yiu MGC Effects of integrated supported employment plus cognitive remediation training for people with schizophrenia and schizoaffective disorders. *Schizophr Res.* (2015) 166:297–303. 10.1016/j.schres.2015.05.013 26044114

[45] Fisher M Nahum M Howard E Rowlands A Brandrett B Kermott A Supplementing intensive targeted computerized cognitive training with social cognitive exercises for people with schizophrenia: an interim report. *Psychiatr Rehabil J.* (2017) 40:21–32. 10.1037/prj0000244 28368179 PMC5380146

[46] Lindenmayer J-P Khan A McGurk SR Kulsa MKC Ljuri I Ozog V Does social cognition training augment response to computer-assisted cognitive remediation for schizophrenia? *Schizophr Res.* (2018) 201:180–6. 10.1016/j.schres.2018.06.012 29910120

[47] Inchausti F García-Poveda NV Ballesteros-Prados A Ortuño-Sierra J Sánchez-Reales S Prado-Abril J The effects of metacognition-oriented social skills training on psychosocial outcome in schizophrenia-spectrum disorders: a randomized controlled trial. *Schizophr Bull.* (2018) 44:1235–44. 10.1093/schbul/sbx168 29267940 PMC6192494

[48] American Psychiatric Association [APA]. *Diagnostic And Statistical Manual Of Mental Disorders.* Washington, DC: American Psychiatric Association (2000).

[49] Juckel G Schaub D Fuchs N Naumann U Uhl I Witthaus H Validation of the personal and social performance (PSP) scale in a German sample of acutely ill patients with schizophrenia. *Schizophr Res.* (2008) 104:287–93. 10.1016/j.schres.2008.04.037 18595665

[50] Birchwood M Smith J Cochrane R Wetton S Copestake S. The social functioning scale the development and validation of a new scale of social adjustment for use in family intervention programmes with schizophrenic patients. *Br J Psychiatry.* (1990) 157:853–9. 10.1192/bjp.157.6.853 2289094

[51] Morosini PL Magliano L Brambilla La Ugolini S Pioli R. Development, reliability and acceptability of a new version of the DSM-IV social and occupational functioning assessment Scale (SOFAS) to assess routine social funtioning. *Acta Psychiatr Scand.* (2000) 101:323–9. 10.1111/j.1600-0447.2000.tb10933.x 10782554

[52] Nemoto T Kashima H Mizzuno M. Contribution of divergent thinking to community functioning in schizophrenia. *Prog Neuro-Psychoph.* (2007) 31:517–24. 10.1016/j.pnpbp.2006.12.001 17218048

[53] Almerie MQ Okba Al Marhi M Jawoosh M Alsabbagh M Matar HE Maayan N Social skills programmes for schizophrenia. *Cochrane Database Syst Rev.* (2015) 2015:CD009006. 10.1002/14651858.CD009006.pub2 26059249 PMC7033904

[54] Vita A Barlati S Ceraso A Nibbio G Ariu C Deste G Effectiveness, core elements, and moderators of response of cognitive remediation for schizophrenia: a systematic review and meta-analysis of randomized clinical trials. *JAMA Psychiatry.* (2021) 78:848–58. 10.1001/jamapsychiatry.2021.0620 33877289 PMC8058696

[55] Cooper RE Laxhman N Crellin N Moncrieff J Priebe S. Psychosocial interventions for people with schizophrenia or psychosis on minimal or no antipsychotic medication: a systematic review. *Schizophr Res.* (2020) 225:15–30. 10.1016/j.schres.2019.05.020 31126806

[56] Morrison AP Pyle M Maughan D Johns L Freeman D Broome MR Antipsychotic medication versus psychological intervention versus a combination of both in adolescents with first-episode psychosis (MAPS): a multicentre, three-arm, randomised controlled pilot and feasibility study. *Lancet Psychiatry.* (2020) 7:788–800. 10.1016/S2215-0366(20)30248-0 32649925 PMC7606914

