# Supplementary material for: Psychoeducation, motivational interviewing, cognitive remediation training, and/or social skills training in combination for psychosocial functioning of patients with schizophrenia spectrum disorders: A systematic review and meta-analysis of randomized controlled trials
Source: Front Psychiatry. 2022 Sep 30;13:899840. doi: 10.3389/fpsyt.2022.899840 (PMC9561245; doi:10.3389/fpsyt.2022.899840)
Supplement: Supplementary file 1 [file Table_1.DOCX]

Supplementary File

Data used for analyses reported in Table 3

| Outcome | Study | Intervention | | | Control | | |
| --- | --- | --- | --- | --- | --- | --- | --- |
|  |  | Mean | SD | n | Mean | SD | n |
| Positive symptoms | Fisher et al., 2017 | 14.97 | 0.85 | 29 | 13.96 | 0.85 | 32 |
|  | Inchausti et al., 2018 | 23.39 | 5.39 | 35 | 23.45 | 5.35 | 33 |
|  | Lindenmayer et al., 2018 | 18.05 | 4.88 | 39 | 17.01 | 3.52 | 39 |
|  | Valencia et al., 2012 | 8.5 | 2.0 | 39 | 10.4 | 4.0 | 34 |
|  | Valencia et al., 2013 | 11.3 | 10.6 | 68 | 14.0 | 7.8 | 51 |
| Negative symptoms | Fisher et al., 2017 | 15.83 | 0.84 | 29 | 14.02 | 0.81 | 32 |
|  | Inchausti et al., 2018 | 17.5 | 4.46 | 35 | 18.85 | 4.03 | 33 |
|  | Lindenmayer et al., 2018 | 18.99 | 3.45 | 39 | 18.69 | 3.53 | 39 |
|  | Valencia et al., 2012 | 10.4 | 3.9 | 39 | 14.6 | 6.3 | 34 |
|  | Valencia et al., 2013 | 11.2 | 7.8 | 68 | 14.9 | 5.1 | 51 |
| General psychopathology | Fisher et al., 2017 | 32.08 | 1.54 | 29 | 30.33 | 1.48 | 32 |
|  | Inchausti et al., 2018 | 34.47 | 10.92 | 35 | 35.12 | 10.23 | 33 |
|  | Valencia et al., 2012 | 21.3 | 4.6 | 39 | 27.6 | 8.1 | 34 |
| Overall score of symptom severity | Au et al., 2015 | 22.49 | 3.38 | 45 | 22.89 | 3.79 | 45 |
|  | Fisher et al., 2017 | 64.16 | 2.71 | 29 | 57.72 | 2.63 | 32 |
|  | Inchausti et al., 2018 | 75.36 | 24.88 | 35 | 77.42 | 23.77 | 33 |
|  | Lindenmayer et al., 2018 | 68.23 | 10.25 | 39 | 67.98 | 11.42 | 39 |
|  | Valencia et al., 2012 | 40.2 | 9.6 | 39 | 52.7 | 15.7 | 34 |
| Speed of processing | Au et al., 2015 | 36.96 | 11.51 | 45 | 44.64 | 39.75 | 45 |
|  | Fisher et al., 2017 | 37.51 | 2.32 | 29 | 41.46 | 2.31 | 32 |
|  | Lindenmayer et al., 2018 | 24.97 | 14.59 | 39 | 22.45 | 13.45 | 39 |
| Visual learning | Au et al., 2015 | 47.73 | 14.23 | 45 | 41.53 | 11.50 | 45 |
|  | Fisher et al., 2017 | 42.22 | 2.25 | 29 | 41.92 | 2.20 | 32 |
|  | Lindenmayer et al., 2018 | 32.63 | 6.84 | 39 | 31.97 | 7.21 | 39 |
| Reasoning and problem solving | Au et al., 2015 | 38.51 | 9.08 | 45 | 39.29 | 9.89 | 45 |
|  | Fisher et al., 2017 | 43.08 | 1.70 | 29 | 44.41 | 1.69 | 32 |
|  | Lindenmayer et al., 2018 | 39.89 | 8.45 | 39 | 37.65 | 7.27 | 39 |
| Attention/vigilance | Au et al., 2015 | 41.77 | 12.41 | 45 | 38.22 | 11.09 | 45 |
|  | Fisher et al., 2017 | 40.25 | 2.08 | 29 | 43.16 | 2.07 | 32 |
|  | Lindenmayer et al., 2018 | 28.18 | 12.11 | 39 | 25.57 | 12.73 | 39 |
|  | Shimada et al., 2018 | -1.88 | 1.06 | 66 | -2.22 | 1.16 | 63 |
